# Multifunctional Properties of Binary Polyrhodanine Manganese Ferrite Nanohybrids—From the Energy Converters to Biological Activity

**DOI:** 10.3390/polym12122934

**Published:** 2020-12-08

**Authors:** Emilia Zachanowicz, Magdalena Kulpa-Greszta, Anna Tomaszewska, Małgorzata Gazińska, Monika Marędziak, Krzysztof Marycz, Robert Pązik

**Affiliations:** 1 Polymer Engineering and Technology Division, Wroclaw University of Technology, 50-370 Wrocław, Poland; malgorzata.gazinska@pwr.edu.pl; 2 Faculty of Chemistry, Rzeszow University of Technology, Aleja Powstańców Warszawy 12, 35-959 Rzeszow, Poland; mkulpa@ur.edu.pl; 3 Department of Biotechnology, Institute of Biology and Biotechnology, College of Natural Sciences, University of Rzeszow, Pigonia 1, 35-310 Rzeszow, Poland; atomaszewska@ur.edu.pl; 4 Faculty of Biology, University of Environmental and Life Sciences Wroclaw, Kożuchowska 5b, 50-631 Wroclaw, Poland; monika.maredziak@gmail.com (M.M.); krzysztofmarycz@interia.pl (K.M.)

**Keywords:** ferrites, polyrhodanine, binary hybrids, energy conversion, biological activity

## Abstract

The PRHD@MnFe_2_O_4_ binary hybrids have shown a potential for applications in the biomedical field. The polymer cover/shell provides sufficient surface protection of magnetic nanoparticles against adverse effects on the biological systems, e.g., it protects against Fenton’s reactions and the generation of highly toxic radicals. The heating ability of the PRHD@MnFe_2_O_4_ was measured as a laser optical density (LOD) dependence either for powders as well as nanohybrid dispersions. Dry hybrids exposed to the action of NIR radiation (808 nm) can effectively convert energy into heat that led to the enormous temperature increase Δ*T* 170 °C (>190 °C). High concentrated colloidal suspensions (5 mg/mL) can generate Δ*T* of 42 °C (65 °C). Further optimization of the nanohybrids amount and laser parameters provides the possibility of temperature control within a biologically relevant range. Biological interactions of PRHD@MnFe_2_O_4_ hybrids were tested using three specific cell lines: macrophages (RAW 264.7), osteosarcoma cells line (UMR-106), and stromal progenitor cells of adipose tissue (ASCs). It was shown that the cell response was strongly dependent on hybrid concentration. Antimicrobial activity of the proposed composites against *Escherichia coli* and *Staphylococcus aureus* was confirmed, showing potential in the exploitation of the fabricated materials in this field.

## 1. Introduction

Superparamagnetic iron oxide nanoparticles (MNPs) have been the subject of great interest in recent years due to their various potential applications in biomedicine [1], electronics [2] or analytical chemistry [3]. Among many, particular attention was paid to well-known magnetic hyperthermia, photothermal therapies or emerging combination of both of them by taking advantage of synergic action of the alternating magnetic field and near infra-red light radiation (NIR) [4,5,6]. One of the reasons is that MNPs offer added value like the ability to guide them by a magnetic field to a specific location through the circulatory system as well as tracking treatment effects with Magnetic Resonance Imaging (MRI) [7]. These advantages make MNPs unique and attractive for tumor targeting therapy [8], targeted drug delivery [9] and imaging [7]. The idea of NIR utilization for heat induction is relatively new and a lot of attention is directed towards this application. It is especially attractive for the shallow and on-skin non-invasive treatment as well as heat triggered drug delivery [10]. Besides the NIR light does not induce damages like UV and penetrates biological systems much deeper due to the minimized water absorption within the so-called biological optical window. Another feature is that NIR laser sources are nowadays more affordable thus costs can be reasonably reduced. For this purpose most commonly used materials are those that have a strong absorbance between 650 and 1350 nm such as metallic nanoparticles, quantum dots or carbon nanotubes characterized by high photothermal efficiency [11]. Recently, we have shown that the manganese-nickel ferrites Mn_1−x_Ni_x_Fe_2_O_4_ MNPs could be used as nanoheaters with very efficient heating ability under NIR exposure [12]. The MnFe_2_O_4_ is a classic example of soft magnetic material [13] but at the same time a narrow band gap semiconductor (1.50–2.23 eV) [14]. Therefore, MnFe_2_O_4_ and other ferrites show high catalytic activity [15]. One of the possible consequences is a cell death induced by Fenton’s reaction generating highly toxic hydroxyl and superoxide radicals [16,17]. Hence, surface blockage is mandatory. Nanoparticle surface functionalization has to deliver sufficient biocompatibility to reduce cytotoxicity, prevent particle aggregation under physiological conditions and minimize MNPs premature elimination by the reticuloendothelial system (RES) [18].

Latterly, we have shown a strategy of ferrites coating by rhodanine oxidative polymerization that can significantly reduce the cytotoxicity of the particles [19]. It is well known that nanohybrid materials with polyrhodanine coating (PRHD) have antibacterial, antifungal, antiviral, antitumor, antidiabetic, anticonvulsant, anti-inflammatory properties [20,21,22]. Thus, in this work, we are primarily focused on the possibility of the heat generation ability of the PRHD@MnFe_2_O_4_ hybrids under the action of NIR laser exposure. Another important goal was to characterize hybrids biological activity during interaction with murine macrophages RAW 264.7, rat osteosarcoma-derived UMR-106 and human adipose derived stromal progenitor cells (ASCs). Moreover, since the PRHD shows significant antibacterial activity the test of hybrids action against standard *E. coli* ATCC 8739 as well as *S. aureus* ATCC 25923 was carried out.

## 2. Materials and Methods

### 2.1. Synthesis of Stock MnFe_2_O_4_ and PRHD@MnFe_2_O_4_ Binary Hybrids

#### 2.1.1. Microwave Driven Solvothermal Synthesis of MnFe_2_O_4_ Nanoparticles

The preparation procedure of the MnFe_2_O_4_ nanoparticles and PRHD@MnFe_2_O_4_ composites was based on a protocol previously described [19,23]. In a typical nanoparticle synthesis 0.6329 g (2.5 mmol) of Mn(acac)_2_ (99.9%, Alfa Aesar, Kandel, Germany) and 1.7658 g (5 mmol) of Fe(acac)_3_ (99.99%, Alfa Aesar) were taken. All operations with metal organic complexes were performed under a protective atmosphere of N_2_ in an acrylic glovebox (P10R250T2—with automatic gas pressure control, GS Glove Box Systemtechnik GmbH, Sömmerda, Germany). The substrates were quantitatively transferred to the Teflon vessel and dissolved in 70 mL of acetophenone (99%, Sigma Aldrich, Poznań, Poland). Afterward, the mixture was placed inside a Magnum V2 microwave reactor (Ertec^®^, Wroclaw, Poland) and processed for 60 min under autogenous pressure of 15 atm at 200 °C. The reaction was stopped and nanoparticles were separated from the black-brown slurry by high-speed centrifugation. Purification of the final product was carried out by washing-centrifugation cycles with the use of 20 mL portions of ethanol (96%, Avantor, Gliwice, Poland). The resulting particles were resuspended in ethanol and served as a stock dispersion for the further polymerization process. The concentration of nanoparticles was evaluated by using the microscale technique and was 23 mg/mL. Part of the stock solution was used for the determination of the basic physicochemical properties of MnFe_2_O_4_ nanoparticles (XRD, TEM, DLS).

#### 2.1.2. Synthetic Protocol for Binary Hybrids Preparation

The oxidation polymerization process was used to fabricate PRHD@MnFe_2_O_4_ binary hybrids. The description refers to the sample denoted further in the text as PRHD@MnFe_2_O_4_ 10%@90% or 1:10. For that purpose 50 mg of rhodanine monomer (C_3_H_3_NOS_2_ 99%, Alfa Aesar) were dissolved in water at 70 °C under vigorous stirring and 100 mL of water nanoparticle dispersion were added containing 50 mg of MnFe_2_O_4_. After 1 h of mixing the polymerization process was initiated by the addition of 50 mg of the FeCl_3_. The mixture was left for 24 h under constant stirring at room temperature. The resulting hybrid material was washed by de-ionized water three times and dried under vacuum at 40 °C for 24 h. The same procedure was adapted for the preparation of other hybrids with different ratios of polymer to nanoparticles. Dispersions of hybrid materials were prepared with given concentrations.

### 2.2. Apparatus

The X-ray powder diffraction technique (XRD) was chosen to determine the structural properties of fabricated stock MnFe_2_O_4_ nanoparticles and PRHD@MnFe_2_O_4_ hybrids. Diffraction patterns were collected on a D8 Advance diffractometer (Bruker, Billerica, MA, USA) equipped with Cu lamp (K_α1_: 1.54060 Å) covering 2Θ range between 10–75°. In order to avoid K_α2_ Ni filter was applied. The crystal phase identification was carried out based on a direct comparison of experimental results with reference standards (JCPDS PDF-2 database). The morphology and particle size estimation of stock nanoparticles was done by means of transmission electron microscope on a CM-20 Super Twin microscope (Philips, Amsterdam, The Netherlands) operating at 200 kV. The standard sample preparation procedure was employed through the deposition of a droplet of nanoparticle suspension (25 µg/mL) on a copper grid covered with the perforated carbon layer and slowly dried under the IR lamp. In the case of hybrid materials, the polymer layer can be damaged under high electron beam energy. Therefore, a Helios Nanolab 660 scanning electron microscope (ThermoFischer Scientific, Waltham, MA, USA) was utilized being able to work under a low voltage regime (2 kV). The droplet of the sample was placed on a carbon tape stuck to the alumina holder and carefully dried under dust protection for 24 h at 25 °C. After that air was evacuated and sample imaging was performed. The hydrodynamic size of particles and hybrids suspensions together with its distribution were measured on a semiautomatic Nanosight NS 500 station (Malvern Panalytical, Malvern, Unitd Kingdom) equipped with a 405 laser source through the DLS technique. The colloidal samples with known concentrations were diluted in ultrapure water and transferred through the peristaltic pump into the measuring chamber. Working concentration giving the most reliable and repeatable results was set at 50 µg/mL by testing different particle concentrations. Thermogravimetric (TGA) analysis was carried out on a TA-300 system (Mettler Toledo, Columbus, OH, USA) within a temperature range of 25–650 °C under a nitrogen atmosphere and with a heating rate of 10 °C/min. Apparatus validation was performed using a standard calcium oxalate reference. Fourier transform infrared spectroscopy technique (FT-IR) was employed for additional materials characterization utilizing a Nicolet iZ10 spectrometer (ThermoFischer Scientific, Waltham, MA, USA) equipped with attenuated total reflection accessory (ATR) within 4000–500 cm^−1^ spectral range. Prior measurement several milliliters of a stock solution containing MnFe_2_O_4_ nanoparticles and binary hybrids were slowly dried and directly placed on the ATR accessory surface (diamond crystal). Specific heat capacities of binary hybrids and PRHD polymer were measured by Differential Scanning Calorimetry (DSC) using the sapphire method [24] on a Mettler Toledo DSC1 system, coupled with a TC 100 intracooler (Mettler Toledo, Columbus, OH, USA). The instrument was fully calibrated using indium (*T_m_* = 156.6 °C, Δ*H_m_* = 28.45 J/g) and zinc (*T_m_* = 419.7 °C, Δ*H_m_* = 107.00 J/g) standards. Samples (~3.5 mg) were measured in 40 µl aluminum pans under a constant nitrogen purge (60 mL/min) from 0 to 50 °C with a heating rate of 10 °C /min. The DSC signal of measured samples was compared with the DSC signal of the sapphire used as a calibration standard of known specific heat. Each curve was corrected (automatic blank curve correction). The DSC experimental data were processed using the generic STAR^e^ software to obtain the specific heat capacity values. Temperature effects on PRHD@MnFe_2_O_4_ were measured with a set-up consisting of continuous wave 808 nm near-infrared (NIR) laser module for stimulation (CNI, Changchun, China) and a T660 thermovision camera (FLIR, Wilsonville, OR, USA). For the laser power validation, an Ophir StarLite laser power meter (Ophir Optronics, Jerusalem, Israel) equipped with a 10 A-PPS beam track thermal sensor (measurable laser power from 20 mW up to 10 W (Ophir Optronics, Jerusalem, Israel) was used. Recorded sample temperature response was analyzed using dedicated ResearchIR software provided by FLIR. Samples were measured by using laser optical density (LOD) within the range of 0.11–0.52 W/cm^2^.

### 2.3. Evaluation of Cytotoxicity of PRHD and PRHD@MnFe_2_O_4_ Hybrids Using Macrophages (RAW 264.7), Osteosarcoma Cells Line (UMR-106), and Stromal Progenitor Cells of Adipose Tissue (ASCs)

For cytotoxicity evaluation of PRHD and PRHD@MnFe_2_O_4_, three different cell types were included in the study: murine macrophages RAW 264.7 and rat osteosarcoma-derived UMR-106 cell lines, as well as human adipose derived stromal progenitor cells (ASCs). Both, RAW 264.7 and UMR-106 cell lines were purchased from American Type Culture Collection (ATCC, Manassas, VA, USA). ASCs were provided by Wroclaw University of Environmental and Life Sciences, Department of Experimental Biology [25]. Cells used for the experiment were isolated from six healthy donors (*n* = 6) with written informed consent. ASCs on passages 3 to 6 were used in all experiments.

After thawing, RAW 264.7, UMR-106, and ASCs cells were grown in sterile Dulbecco’s modified eagle’s medium DMEM with high glucose (Sigma Aldrich) supplemented with 10% fetal bovine serum (FBS, Sigma-Aldrich) and 1% penicillin-streptomycin solution (Sigma-Aldrich), following the provided protocols. All cells were incubated at 37 °C in a humidified 5% CO_2_ atmosphere.

For the analysis of proliferation activity, cells were inoculated into 24-well plates at initial concentration 2 × 10^4^ per well and suspended in a 0.5 mL of culture medium per well. After 4 h pre-incubation, PRHD and PRHD@MnFe_2_O_4_ hybrids diluted in water were added to the culture medium in the following concentrations: 50; 10 and 1 µg/mL. Control cells were exposed to the diluent only. Proliferative activity of tested nanoparticles was investigated after 24 and 48 h of incubation using a resazurin-based assay (TOX-8, Sigma Aldrich) according to the manufacturer’s instructions. Briefly, culture media were replaced with a medium containing 10% of resazurin-based dye and incubated for two hours. Afterward, the supernatants were collected and light absorbance at 600 nm of wavelength, with a distraction of 690 nm was measured using SPECTRO StarNano (BMG Labtech, Jozefow, Poland). The cell number, as well as, proliferation factor (PF) were calculated concerning the standard curve as previously described [26,27]. Cells’ morphology was observed under an inverted light microscope (AxioObserverA1, Zeiss, Warsaw, Poland) and photographs were taken after 48 h using a PowerShot digital camera (Canon, Warszawa, Poland).

### 2.4. Microbiological Sensitivity of PRHD and PRHD@MnFe_2_O_4_ Hybrids on Escherichia coli ATCC 8739 and Staphylococcus aureus ATCC 25923 Bacteria

To evaluate the microbiological activity of PRHD and PRHD@MnFe_2_O_4_ hybrids, obtained nanoparticles were incubated with the following bacteria’s strains: *Escherichia coli* ATCC 8739 and *Staphylococcus aureus* ATCC 25923. Bacteria were cultivated in Muller-Hinton Broth (Sigma-Aldrich) at 37 °C for 24 h. Bacteria samples were suspended in a test tube containing nutrient broth at a concentration of 2.0 × 10^8^ colony-forming units (CFU/mL) by adjusting the turbidity equivalent to McFarland 0.5 standard. The final concentrations were confirmed by agar plating. The same bacterial suspensions were used for every repetition in the course of the study. The Kirby-Bauer diffusion technique was applied for the investigation of the antimicrobial activity of tested nanoparticles. Briefly, within 15 min after adjusting the turbidity of the bacteria suspension was inoculated on plates by a sterile non-toxic swab. Afterward, standard paper discs with a diameter of 10 mm (Whatman Maidstone, United Kingdom), with PRHD and PRHD@MnFe_2_O_4_ hybrids were placed on the agar surface. After 18 to 20 h incubation with PH the inhibition zones were measured. In order to determine the antibacterial activity of the experimental nanoparticles, 100 µL of the suspension was added to 900 µL of nanoparticles and incubated for 24 h as previously described. As a control for investigated nanoparticles, Muller Hinton Broth instead has been used. After 24 h of the incubation period, bacteria’s suspensions were prepared in the following dilution 10^−3^, 10^−4^, 10^−5^, 10^−6^, 10^−7^, 10^−8^ as described previously [19]. Subsequently, 100 µL of each dilution was transferred on agar plates in duplicate. Finally, antibacterial activity was performed using the viable plate count method by counting the colonies more than 15–150 colonies each.

## 3. Results

### 3.1. Physicochemical Properties of Binary PRHD@MnFe_2_O_4_ Hybrids

**Figure 1:**
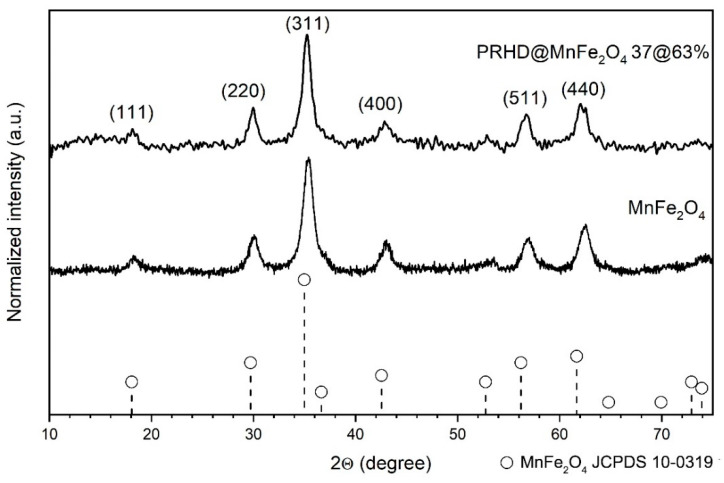
Comparison of the XRD patterns of stock MnFe_2_O_4_ nanoparticles with PRHD@MnFe_2_O_4_ hybrid material.

Structural properties of the PRHD@MnFe_2_O_4_ composites were elucidated using the XRD technique through direct comparison of obtained diffraction patterns for stock nanoparticles and hybrids with the reference MnFe_2_O_4_ standard from the JCPDS database (pattern no. 10-0319). Both materials show reflection peaks located at the same angles and with comparable intensities. No other diffraction peaks that could correspond with impurities were detected (Figure 1). Thus, it was assumed that formation of the polymeric layer on nanoparticles during synthesis does not induce any chemical transformation of the nanoparticles. However, an extra feature was observed namely the appearance of a broad bump at the range of low 2Θ angles (below 15°).

**Figure 2:**
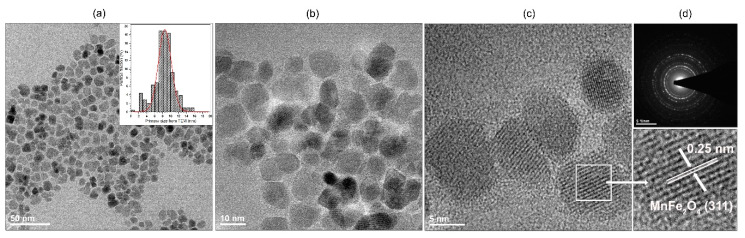
TEM (**a**,**b**), HR-TEM (**c**) and SAED (**d**) images of the MnFe_2_O_4_ stock nanoparticles. The bottom part of (**d**) presents exposed (311) lattice fringes of the MnFe_2_O_4_.

This is treated as an indication of the presence of PRHD since the polymer layer is amorphous and shows no long range order. The mean crystallite size (*D*) was calculated by using the well-known Scherrer equation:

(1) $$D=\frac{k\lambda }{\cos\Theta \sqrt{\beta^{2}-\beta_{0}^{2}}},$$

where *β*_0_ is apparatus broadening (0.05°); *β* stands for full width at half maximum; θ maximum of peak position taken for calculation (half of the 2Θ); *k* is a constant, here equals to 0.9 due to the assumption of particle spherical shape and *λ* is the exact X-ray wavelength source (Cu—1.5406 Å). The advantage of this approach is that it gives a quick but rough idea regarding the crystallite size. The mean size of the stock MnFe_2_O_4_ particles is 7 nm while the PRHD covered material is 12 nm. If one would consider a calculation error then both values are practically the same. Usually, precise size evaluation involves the usage of more advanced techniques, for instance TEM, whereas the determination of particle diameter in a suspension requires DLS. Therefore, TEM images were taken in order to provide a more accurate particle size estimation, distribution, and morphology. TEM in combination with SAED allows for confirmation of the product structural properties from the area of interest (see Figure 2). We noticed that there is a very good correspondence between TEM mean particle size, being 8.3 nm, with the XRD data. Thus, it can be concluded that each particle is comprised of single and separate crystallite. In addition, the inset in Figure 2 shows that particle distribution is fairly narrow while the morphology of the product is rather irregular. In general, the chosen synthetic approach [24] allows for the preparation of hydrophilic and relatively non-agglomerated particles with an open surface for further functionalization. Sufficient colloidal stability is due to the presence of adsorbed organic ligands during the fabrication process [5]. Besides, the hydrophilic character of the MnFe_2_O_4_ nanoparticles is sought as an advantage since the oxidative polymerization process runs under strong hydrolytic conditions [19]. In principle, this feature assures the homogenous distribution of particles within the reaction mixture. The SAED shows typical diffraction for polycrystalline materials and consists of a dot-ring image of reflections with intensities and plane distances corresponding with the MnFe_2_O_4_ reference standard. HRTEM picture shows that the particles tend to expose well-defined crystallographic planes which can be treated as an indication of the high crystallinity of stock particles.

**Figure 3:**
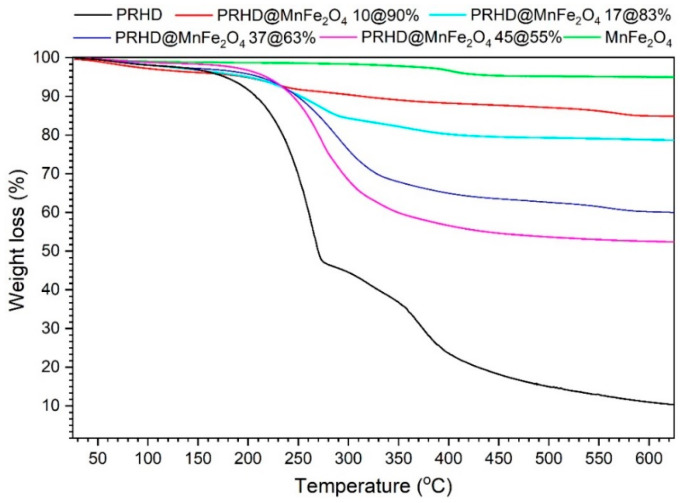
TGA analysis of PRHD@MnFe_2_O_4_ hybrid materials.

Analysis of the thermal behavior of PRHD@MnFe_2_O_4_ binary hybrids was based on the TGA measurement conducted under inert nitrogen atmosphere at a temperature in the range of 25–650 °C (Figure 3). As was previously reported [28] the decomposition of the PRHD polymer is a complex and multistep process.

Initially (up to 110 °C) release of the moisture occurs, whereas between 110–290 °C degradation of the PRHD chain and depolymerization progresses. The 290–425 °C range is attributed to the 2nd and 3rd degradation steps of PRHD [28]. In the case of the hybrid materials, decomposition proceeds similarly but the amount of released organic material is different and depends on the quantity of PRHD deposited on a nanoparticle surface. It is worth noting that the MnFe_2_O_4_ nanoparticles shift the decomposition temperature of the polymer to higher ones, enhancing PRHD thermal stability. This is also consistent with previous reports on PRHD hybrids with cores containing Fe_3_O_4_ [28] and CoFe_2_O_4_ [19] nanoparticles. The content (%) of the polymer was calculated with respect to the MnFe_2_O_4_ nanoparticles and is as follows PRHD@MnFe_2_O_4_ 10%@90%, PRHD@MnFe_2_O_4_ 17%@83%, PRHD@MnFe_2_O_4_ 37%@63%, and PRHD@MnFe_2_O_4_ 45%@55%, respectively.

**Figure 4:**
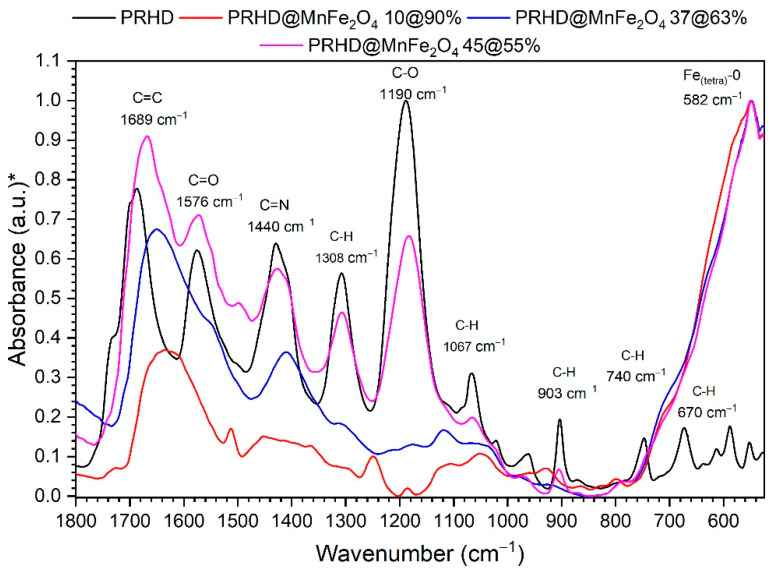
FT-IR-ATR spectra of the reference PRHD (red line) and hybrids PRHD@MnFe_2_O_4_. (*) normalized absorbance for comparison purposes.

To prove the effect of the MnFe_2_O_4_ surface coverage with different ratios of the PRHD polymer FT-IR-ATR spectra were recorded (Figure 4). As the reference pure PRHD fabricated by the same synthetic protocol was used. The measured PRHD spectra reveal the presence of characteristic band groups at 1684, 1576, 1440, 1187 cm^−1^ associated with the C=C, C=O, C=N^+,^ and C–O^−^ vibrations. All of them were ascribed to the polymer and are in good agreement with the literature data [29]. The aforementioned vibration modes are clearly identifiable in the spectra of the hybrid materials. It is especially visible for the sample containing 45% of the PRHD (the thickest layer). Upon reduction of the polymer ratio/thickness, the band intensities decrease significantly, but still, the peak at 1684 cm^−1^ remains quite intense allowing for detection of the PRHD presence. Except that, one can observe a highly intense band located at around 590 cm^−1^. The nature of this mode is associated with vibrations of the Fe–O bonds at the tetrahedral site that corresponds with the ferrite/spinel structure. Thus, it can be treated as a fingerprint of the MnFe_2_O_4_ phase in the IR spectra [30]. We would like to emphasize that even though the oxidative polymerization requires the presence of FeCl_3_ as an oxidizer, the IR spectra of pure PRHD is free of vibrations linked to the presence of any iron oxide material. It means that the purification steps are effective and there is no formation of additional iron oxides during the polymerization process. However, the presence of some iron cations cannot be completely excluded since the PRHD contains several possible coordination sites located on heteroatoms.

**Figure 5:**
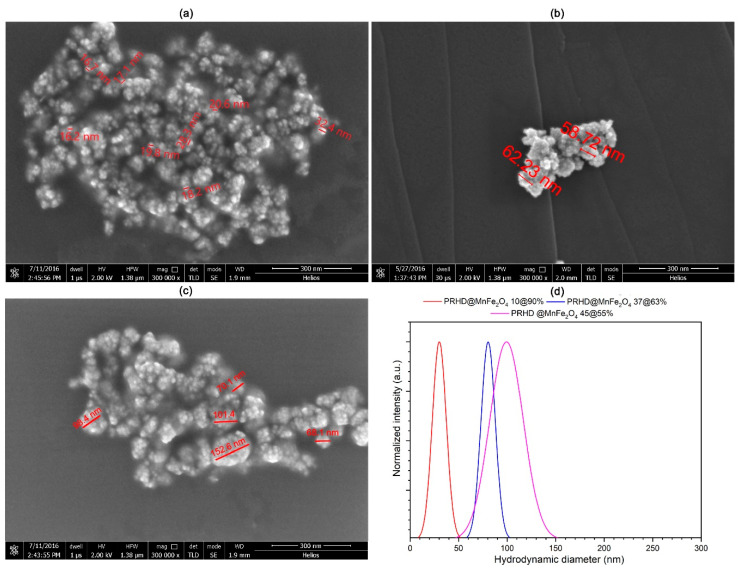
SEM images (**a**–**c**) and hydrodynamic size (**d**) of hybrid materials with different ratios (%) of PRHD vs. MnFe_2_O_4_ (**a**) 10/90, (**b**) 37/63, and (**c**) 45/55.

The particle diameter of the binary hybrids was estimated by using DLS technique and verified through SEM microscopy intended to work with soft materials (see Figure 5). It is worth noting that the hybrid particle hydrodynamic dimensions depend strongly on the PRHD amount. Size of composites changes progressively from around 20 ± 2.6 nm for the PRHD@MnFe_2_O_4_ 10%@90%, 62 ± 20 nm, for the PRHD@MnFe_2_O_4_ 37%@63%, and 110 ± 50 nm for the PRHD@MnFe_2_O_4_ 45%@55%, respectively. SEM images confirm that observation, though increase of the monomer ratio affects the thickness of the PRHD layer. It is therefore anticipated that this will chnage the physicochemical properties of hybrids. The measurement of the hydrodynamic size pointed out the preservation of the particle diameters. Thus, it is obvious that the polymer coating efficiently reduces the surface energy of nanoparticles and is able to deliver sufficient stability of the PRHD@MnFe_2_O_4_ composites in a suspending medium.

### 3.2. Effectiveness of Heat Induction by PRHD@MnFe_2_O_4_ Binary Hybrids

**Figure 6:**
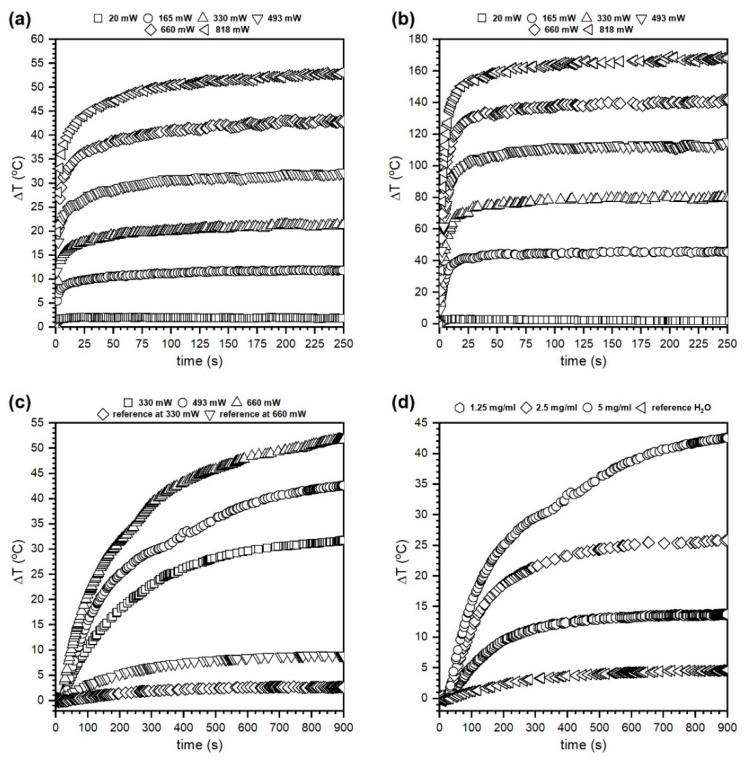
Heat induction of the PRHD and PRHD@MnFe_2_O_4_ hybrids under stimulation of 808 nm NIR laser for dry powders (upper panel) and colloidal suspensions (bottom panel). The (**a**,**b**) plots represent the behavior of PRHD and PRHD@MnFe_2_O_4_, (**c**) shows the power dependence of PRHD@MnFe_2_O_4_ dispersion (5 mg/mL), and (**d**) concentration dependence of PRHD@MnFe_2_O_4_ suspensions (LOD 0.31 W/cm^2^), respectively. Reference measurements done on pure water (**c**)—for 0.21 and 0.42 W/cm^2^ and (**d**) 0.31 W/cm^2^.

NIR laser radiation (808 nm) conversion into heat was measured for the PRHD@MnFe_2_O_4_ 37%@63% powders and nanoparticle suspensions as a function of the laser optical density (LOD, 0.11–0.52 W/cm^2^) and nanoparticle hybrid concentration (1.25–5 mg/mL) in a water medium. As a reference material, water and PRHD heating capabilities were measured for minimum and maximum laser powers used in the experiments to evidence the composite potential for the heat generation (see Figure 6). Since both PRHD and hybrid powders have shown an extreme increase of temperature, the duration of each experiment was limited to the 250 s (top panels in Figure 6). In the case of hybrid suspension much slower heat exchange occurs between nanoparticles and water medium thus experiment time was extended to 900 s to achieve a reasonable plateau and limit laser exposure to an absolute minimum (bottom panels in Figure 6).

**Figure 7:**
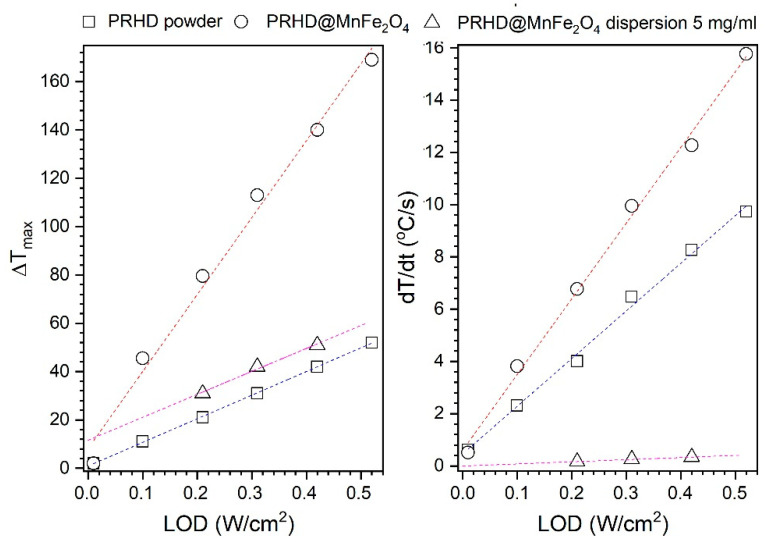
Dependence of the LOD on *ΔT_max_* and *dT/dt* for the dry PRHD, PRHD@MnFe_2_O_4_ hybrids, and colloidal suspension of PRHD@MnFe_2_O_4_ (5 mg/mL) under stimulation of 808 nm NIR.

We would like to emphasize that right after the polymerization and purification process the solution containing PRHD was dark yellow. The sample color is a consequence of the absorption of chromophores (heteroatoms, π bonds, etc.) in the molecular structure. Further hybrid extraction from the reaction medium combined with slow drying leads to color deepening (solvent removal). The UV-VIS absorption spectra recorded by Yang et al. [31] shows the presence of a broad absorption band with a long shoulder ranging above 800 nm with relatively high absorbance. Therefore, the heat induction was tested on the PRHD dry polymer (Figure 6a) as a function of the laser output power (20–818 mW, corresponding LOD was within (0.01–0.52 W/cm^2^). One can note that the PRHD absorbs and converts light into heat energy achieving Δ*T* of 52 °C (above 70 °C since measurement started from 23 °C) for the maximum LOD of 0.52 W/cm^2^. In the case of the composite PRHD@MnFe_2_O_4_ (Figure 6b) laser exposure leads to an enormous temperature increase that exceeds Δ*T* 170 °C (above 190 °C) for maximum LOD (0.52 W/cm^2^) and even at low optical densities (0.1 W/cm^2^) the sample heats up to 68 °C (Δ*T* was 45.4 °C). Higher LODs were not tested since there was a significant risk of polymer layer damage due to the generation of temperature in the range close to the decomposition conditions. The ability to light conversion into heat on composite material is due to the substantial light absorption by MnFe_2_O_4_ nanoparticles [12,32]. The main mechanism of energy dissipation will rely on the dominating character of non-radiative processes (net phonons). The temperature effect on dry materials is very rapid. After 30 s of light irradiation maximum temperature was reached. Based on the results of effective heat induction on dry binary hybrids the water suspension containing 5 mg/mL of polymer coated nanoparticles was prepared and measured as a function of laser output power (Figure 6c). Afterward, several dilutions were made to estimate the possibility of temperature control through the limitation of nanoheaters concentration (Figure 6d). Due to the high specific heat capacity of water (4.186 J/g°C) and relatively low number of particles duration of the experiment was set to 900 s (15 min). Each experiment started at 23 °C. As can be seen in Figure 6c the dispersion containing 5 mg/mL is very responsive to the external stimulation. For the highest laser power of 660 mW (LOD 0.42 W/cm^2^) Δ*T* was 51 °C (74 °C) and the lowest 330 mW (0.21 W/cm^2^) was Δ*T* 31 °C (44 °C), respectively. Therefore, the concentration dependence (Figure 6d) was measured for the laser power of 493 mW that corresponds to 0.31 W/cm^2^. This value is the maximum approved for irradiation of biological systems and considered as safe laser dosage [33]. It is worth noting that the 5 mg/mL of hybrid material can generate Δ*T* of 42 °C (65 °C) which is too high, but dilution to 2.5 mg/mL leads to Δ*T* of 25 °C (48 °C) whereas 1.25 mg/mL of nanoparticles gives Δ*T* of 14 °C (38 °C). Therefore, it was assumed that it is possible to control medium temperature by optimization of the heating effect through concentration of nanoheaters. Figure 7 presents the dependence of the LOD on the maximum value of Δ*T* and *dT/dt* temperature increase rate (°C/s) estimated from the linear fit for the first tens of seconds of an experiment for dry PRHD, PRHD@MnFe_2_O_4_ and PRHD@MnFe_2_O_4_ hybrid dispersion with a concentration of 5 mg/mL.

Both parameters depend on the applied LOD and change linearly with the LOD increase. It is interesting to note that the *dT/dt* values characterizing colloids are significantly lower but this is anticipated behavior since the particles have to dissipate heat effectively and warm up a medium with high specific heat capacity. We would like to emphasize that 0.5% of the total mass are nanoheaters, so the temperature rate must be much smaller in comparison to dry materials.

**Figure 8:**
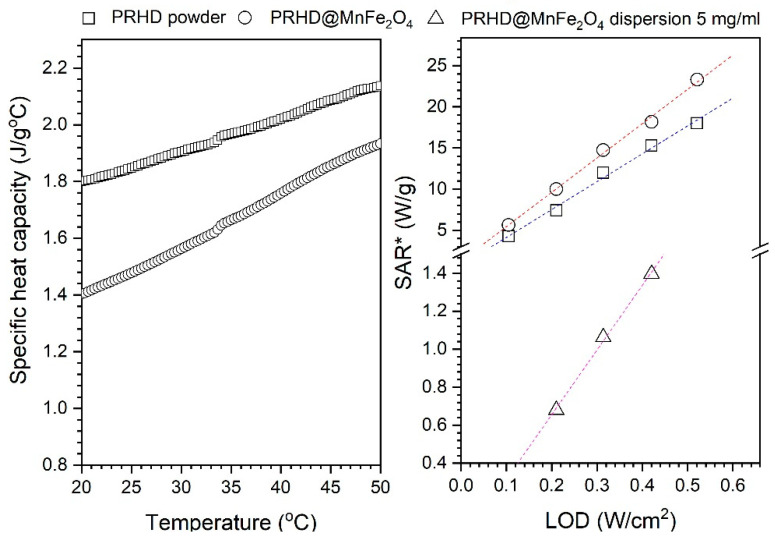
Specific heat capacities of PRHD and PRHD@MnFe_2_O_4_ samples measured at the temperature range of 20–50 °C (**left**) and LOD dependence on *SAR* values (**right**). *SAR calculated for the whole system.

The effectiveness of the heat induction can be estimated by calculation of the specific absorption rate (*SAR* units W/g) almost directly from a measurement of the sample temperature behavior using the following formula:

(2) $$SAR=C\frac{dT}{dt},$$

where *C* is the specific heat capacity of a sample taken from DSC experiment or tabularized data (J/g°C) and *dT/dt* is the slope of the heating curve fitted for the first seconds of the measurement with help of a linear model (°C/s). Since the value of specific heat capacity of PRHD is not given in the literature and the same stands for the PRHD@MnFe_2_O_4_ hybrid we decided to measure both of them using the DSC technique (Figure 8). The calculated specific heat capacities for PRHD and PRHD@MnFe_2_O_4_ taken for *SAR* calculation were 1.85 and 1.48 J/g°C, respectively. In the case of the colloidal suspension, the specific heat capacity of water was taken since the contribution of the MnFe_2_O_4_ specific heat capacity is negligible at such nanoparticle concentrations (0.5% in respect to the total sample mass). Implantation of the Equation (2) means that we calculate all *SAR* values for the whole systems to be able to compare heat induction ability upon change of the environments. The results of *SAR* calculation for measured materials were gathered in Table 1. As one can see *SAR* changes clearly with the LOD increase from 4.3 to 18 W/g for PRHD and 5.6 up to 23.3 W/g for hybrid showing that there is a significant effect of the MnFe_2_O_4_ presence in the composite material. In the case of the colloidal suspensions of the PRHD@MnFe_2_O_4_ *SAR* drops dramatically from 1.4 to 0.68 W/g depending on LOD and from 1.06 to 0.26 W/g upon concentration decrease. This effect is expected since the nanoheaters concentration is now very limited and the medium specific heat capacity changes to the high value (4.185 J/g°C). In the literature when one considers colloidal suspensions, it is more common to show the *SAR* value in respect to the nanoparticle content, therefore modified formula can be used:

(3) $$SAR=\frac{Cm_{colloid}}{m_{NPs}}\frac{dT}{dt},$$

where *C* is again the specific heat of water (4.185 J/g°C), *m_colloid_* (g) represents the mass of the whole colloid used in measurement (0.1 g), *m_NPs_* is the total mass of the nanohybrids (restricted to the phase with heating ability) in grams and *dT/dt* with the same meaning as in formula (2). In that way, *SAR* for the colloidal hybrids will change from 136 to 279 W/g depending on the LOD used in the experiment for the concentration equal to 5 mg/mL. However, the use of Equation (3) for comparison of samples in a different state—dry powders and suspensions has no further sense. Nonetheless, fabricated hybrid materials can be sought as an interesting multifunctional platform for different biological related applications where stimulation of important processes through contactless temperature control are necessary i.e., regenerative medicine, cancer treatment or other diagnostic (contrast agent), protein separation, etc. Direct comparison of the energy conversion ability into heat with other hybrids containing magnetite nanoparticles gives quite comparable results [6]. However one has to remember that most often different stimulation are used, namely alternating magnetic field (AMF). Thus, mechanisms involved differ in nature significantly. Combining polymers with magnetic nanoparticles into an organic-inorganic hybrid can be beneficial in many aspects. First of all, polymer, as PRHD, can alter biocompatibility of the composite, provide additional reactive sites for interaction with biomolecules (markers, drugs, antibodies, etc.) thus serve as a specific carrier. Whereas the presence of magnetic material can induce via interaction with AMF, NIR, or both stimuli temperature response needed to release biologically active substances, support temperature controlled regenerative processes (diathermia), induce cell death through hyperthermia or under extreme conditions cell ablation [4,6]. The novelty of our proposal can be also underlined by the utilization of the PRHD itself as a model for studies and engineering of new drugs since its molecular structure contains accessible and reactive sites.

### 3.3. Evaluation of Cytotoxicity of PRHD and PRHD@MnFe_2_O_4_ Hybrids Using Macrophages (RAW 264.7), Osteosarcoma Cells Line (UMR-106), and Stromal Progenitor Cells of Adipose Tissue (ASCs)

**Figure 9:**
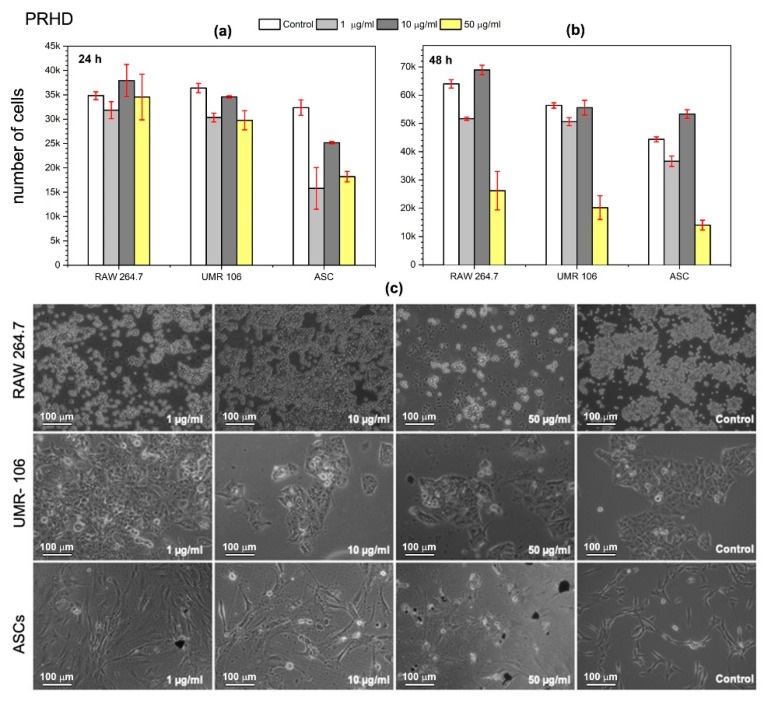
Effect of in vitro PRHD stimulation of macrophages (RAW 264.7), osteosarcoma (UMR-106), and stem (ASCs) cells proliferative activity as a function of concentration (**a**) after 24 h and (**b**) after 48 h of exposure. Panel (**c**) shows morphology changes of macrophages (RAW 264.7), osteosarcoma-derived cells (UMR-106), and stem cells (ASCs) after 48 h in vitro exposure to PRHD nanoparticles and control.

**Figure 10:**
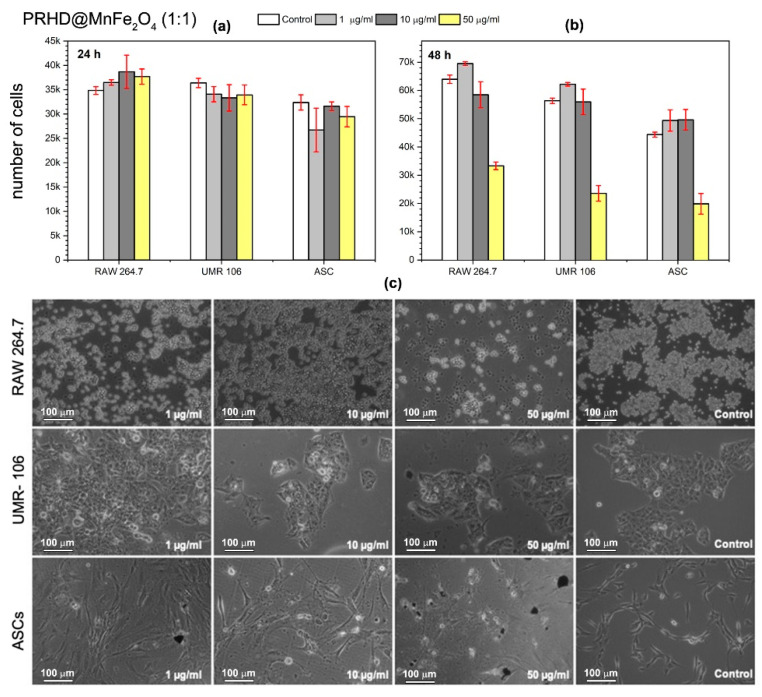
Effect of in vitro PPRHD@MnFe_2_O_4_ (1:1) stimulation of macrophages (RAW 264.7), osteosarcoma (UMR-106), and stem (ASCs) cells proliferative activity as a function of concentration (**a**) after 24 h and (**b**) after 48 h of exposure. Panel (**c**) shows morphology changes of macrophages (RAW 264.7), osteosarcoma-derived cells (UMR-106), and stem cells (ASCs) after 48 h in vitro exposure to PRHD nanoparticles and control.

**Figure 11:**
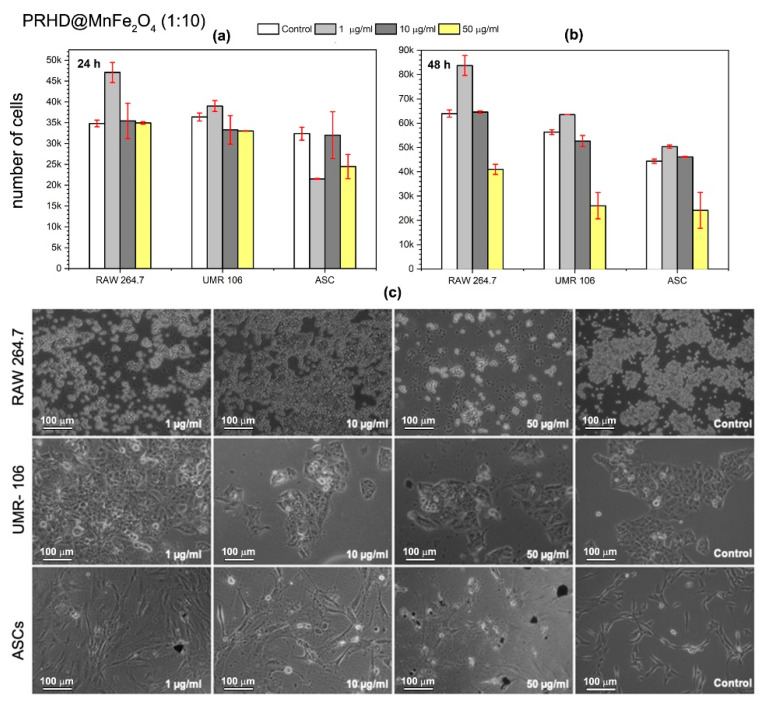
Effect of in vitro PPRHD@MnFe_2_O_4_ (1:10) stimulation of macrophages (RAW 264.7), osteosarcoma (UMR-106), and stem (ASCs) cells proliferative activity as a function of concentration (**a**) after 24 h and (**b**) after 48 h of exposure. Panel (**c**) shows morphology changes of macrophages (RAW 264.7), osteosarcoma-derived cells (UMR-106), and stem cells (ASCs) after 48 h in vitro exposure to PRHD nanoparticles and control.

In the present study, we investigated whether the PRHD and PRHD@MnFe_2_O_4_ hybrids affect proliferative activity as well as the morphology of three different cell types (see Figure 9, Figure 10 and Figure 11). We decided to test nanoparticles to assess their effect on cells modulating inflammatory reaction (macrophages), tumor (osteosarcoma), and stem progenitor cells (ASCs). The latter ones are recently extensively investigated since stem cells possess unique cytophysiological and pro-regenerative properties. We have shown, that both PRHD and PRHD@MnFe_2_O_4_ hybrids can regulate the cytophysiological activity of cells in a dose dependent manner. PRHD nanoparticles significantly reduced the viability of macrophages, osteosarcoma-derived cells, and ASCs when used in higher concentrations (10 and 50 µg/mL). Simultaneously with reduced proliferative activity, cells also lost their typical morphology and presented apoptotic-like phenotype (see Figure 9).

The lowest dose (1 µg/mL) did not affect the investigated cells’ metabolic activity after the first 24 h of exposure. Interestingly, the PRHD when incorporated in a concentration of 1 µg/mL stimulated both RAW 264.7 and UMR-106 cells for increased proliferative activity. In addition, the lowest PRHD concentration, besides improving the proliferative activity of macrophages and osteosarcoma, cells did not negatively affect their morphology and growth pattern. In contrast, ASCs exposed to each concentration of PRHD lost their fibroblast-like morphology and reduced proliferative activity.

The PRHD@MnFe_2_O_4_ in ratio 1:1 (45%@55%) and 1:10 (10%@90%) affected all investigated cells’ viability and morphology in a dose dependent manner (Figure 10 and Figure 11). It was shown, that hybrid nanoparticles effectively improved cellular proliferative activity in the first 24 h. Macrophages and tumor cells maintained their typical morphology. Nevertheless, ASCs exposed even to the lowest concentration of PRHD@MnFe_2_O_4_ lost their proliferative potential and shown apoptotic phenotype. Interestingly, PRHD@MnFe_2_O_4_ in ratio 1:10 (10%@90%), in a concentration 50 µg/mL when incorporated into both macrophages and osteosarcoma cell cultures reduced their activity, significantly changed morphology, and slightly reduced ASCs activity. This sheds a promising light for their potential application as anti-inflammatory as well as anti-tumor factors, which might be used in a broad spectrum of biomedical related fields.

### 3.4. Microbiological Sensitivity of PRHD and PRHD@MnFe_2_O_4_ Hybrids on Escherichia coli ATCC 8739 and Staphylococcus aureus ATCC 25923 Bacteria

The antibacterial efficacy of the nanoparticles and hybrids using the agar diffusion method is presented in Table 2. Test of inhibition zones is highly qualitative and if the bacteria are susceptible to the used materials an area appears where bacteria cannot grow. Statistical significance was determined using the one way ANOVA with Dunnett post hoc test (Prism5.04, GraphPad Software, San Diego, CA, USA). *p* < 0.05 was considered statistically significant. One can note that the PRHD shows pronounced antibacterial properties against *E. coli* and *S. aureus* while hybrids activity depends on the polymer amount and was comparable with PRHD action for a 1:1 ratio. Kirby-Bauer disk technique was additionally confirmed by the CFU test (see Table 3). We observed a meaningful reduction of the number of viable bacteria in all studied materials which are dependent on the content of the polymer and increase with the amount of PRHD. Therefore, the antibacterial potential of proposed binary hybrids might be used in the bactericidal applications where the PRHD plays the role of antibacterial agent whereas MnFe_2_O_4_ due to magnetic properties can facilitate material collection after the disinfection process was finished. Thus it could assure multiple usages of hybrids in mentioned applications.

## 4. Conclusions

Binary hybrids of PRHD@MnFe_2_O_4_ with different thicknesses of the polymeric shell were fabricated using the oxidation polymerization process. It was shown that the average particle size does not increase significantly upon nanoparticle suspension in water media, thus the PRHD can act as a modifying agent preventing particle aggregation (hydrodynamic size between 20 and 90 nm depending on the polymer to MNPs ratio). The MnFe_2_O_4_ magnetic ferrite particles play a multifunctional role as an important ingredient in improved thermal resistance of the polymer, heat enhancement element as well as magnetic collecting agent facilitating hybrid separation from the suspending medium. The specific heat capacity of the PRHD polymer was measured for the first time within the temperature range of 20 to 50 °C (1.4–2.15 J/g°C). We observed that the PRHD polymer due to its absorption ability within the near IR spectral region can also convert energy into heat. However, the addition of MNPs increased the overall heating effectiveness greatly. The temperature response of the hybrids in a powder state for the action of NIR 808 nm laser at different LODs is efficient and rapid (*dT/dt* changes from 3 up to almost 16 °C/s, depending on the LOD). Composite dispersion in water leads to a drop of heating capabilities due to the high specific capacity of the medium. The effect can be improved either by LOD or concentration change.

We have shown, that the PRHD and PRHD@MnFe_2_O_4_ become an interesting source of nanoparticles, exhibiting immunomodulatory effect, and antimicrobial activity. Interestingly, obtained hybrid materials are characterized by antitumorigenic effects. Further research is required for an understanding of the mechanisms involved in the hybrids related cellular action. We would like to underline that the energy conversion on PRHD@MnFe_2_O_4_ hybrids has never been performed before as well as there is no single data on the temperature behavior of this polymer alone. None has proposed and considered such composite as a multifunctional platform for biological applications. We are convinced that this versatile composite is an interesting material that should find its possible implementation in the bio-related field.

## Figures and Tables

**Table 1:** Specific absorption rate (*SAR*) calculated for dry PRHD and PRHD@MnFe_2_O_4_ powders and colloidal suspensions of hybrids.

Sample	LP (mW)	LOD (W/cm^2^)	*dT/dt* (°C/s)	*SAR* (W/g)
PRHD	165	0.11	2.31	4.3
	330	0.21	4.00	7.4
	493	0.31	6.47	12.0
	660	0.42	8.25	15.3
	818	0.52	9.72	18.0
PRHD@MnFe_2_O_4_	165	0.11	3.81	5.6
	330	0.21	6.77	10.0
	493	0.31	9.95	14.7
	660	0.42	12.26	18.1
	818	0.52	15.76	23.3
Dispersion power dependence (5 mg/mL)				
PRHD@MnFe_2_O_4_	330	0.21	0.16	0.68
	493	0.31	0.25	1.06
	660	0.42	0.33	1.40
Dispersion concentration dependence				
1.25 mg/mL	493	0.31	0.06	0.26
2.5 mg/mL	493	0.31	0.17	0.72
5 mg/mL	493	0.31	0.25	1.06

**Table 2:** Antibacterial activity of PRHD@MnFe_2_O_4_ hybrids against pathogenic bacteria (inhibition of growth expressed as the diameter of inhibition zone).

Sample	*E. coli* Inhibition Zone (mm)		*S. aureus* Inhibition Zone (mm)	
PRHD	32	29	31	29
PRHD@MnFe_2_O_4_ (1:1)	28	27	29	32
PRHD@MnFe_2_O_4_ (1:10)	24	23	22	19
Control	0	1	0	1

**Table 3:** Antibacterial assessment of different PRHD@MnFe_2_O_4_ binary hybrids.

Sample	*E. coli* (CFU/mL)	*S. aureus* (CFU/mL)
PRHD	5.0 × 10^2^	1.1 × 10^2^
PRHD@MnFe_2_O_4_ (1:1)	1.2 × 10^2^	1.8 × 10^2^
PRHD@MnFe_2_O_4_ (1:10)	1.2 × 10^3^	1.3 × 10^3^
Control	1.2 × 10^5^	1.3 × 10^5^
